# 

**Published:** 1867-06

**Authors:** 


### CHICAGO MEDICAL EXAMINER.

**N. S. DAVIS, M.D., EDITOR.**

A MONTHLY JOURNAL

DEVOTED TO THE

EDUCATIONAL, SCIENTIFIC, AND PRACTICAL INTERESTS

OF THE

MEDICAL PROFESSION.

The Examiner will be issued during the first week of each month, commencing with January, 1860. Each number will contain 64pages of reading [illegible]atter, the greater part of which will be filled with such contents as will [illegible]irectly aid the practitioner in the daily practical duties of his profession.

To secure this object fully, we shall give, in each number in addition to [illegible]rdinary original articles, and selections on practical subjects, a faithful re[illegible]ort of many of the more interesting cases presented at the Hospitals and [illegible]ollege Cliniques. While aiming, however, to make the Examiner eminently [illegible]ractical, we shall not neglect either scientific, social, or educational interests, [illegible]f the profession. It will not be the special organ of any one institution, [illegible]ociety, or clique; but its columns will be open for well-written articles from [illegible]ny respectable member of the profession, on all topics legitimately within the [illegible]omain of medical literature, science, and education.

Terms, $3.00 per annum, invariably in advance.

